# Electrocardiographic assessments and cardiac events after fingolimod first dose – a comprehensive monitoring study

**DOI:** 10.1186/s12883-016-0789-7

**Published:** 2017-01-18

**Authors:** Volker Limmroth, Tjalf Ziemssen, Michael Lang, Stephan Richter, Bert Wagner, Judith Haas, Stephan Schmidt, Kathrin Gerbershagen, Christoph Lassek, Luisa Klotz, Olaf Hoffmann, Christian Albert, Katrin Schuh, Monika Baier-Ebert, Guillaume Wendt, Heinke Schieb, Susanne Hoyer, Ralf Dechend, Wilhelm Haverkamp

**Affiliations:** 1 Department of Neurology, Cologne General Hospitals, University of Cologne, Cologne, Germany; 2 Center of Clinical Neuroscience, University Clinic Carl Gustav Carus Dresden, Dresden, Germany; 3 NTD Study Group, Neurologische Praxis, Ulm, Germany; 4 NeuroMVZ, Stuttgart, Germany; 5 Jewish Hospital Berlin, Berlin, Germany; 6 NTD Study Group, Neurologische Gemeinschaftspraxis, Bonn, Germany; 7 Neurologische Gemeinschaftspraxis Kassel und Vellmar, Kassel, Germany; 8 Department of Neurology, University of Muenster, Muenster, Germany; 9 St. Josefs-Krankenhaus Potsdam-Sanssouci, Potsdam, Germany; 10 Novartis Pharma GmbH, Nuremberg, Germany; 11 Experimental and Clinical Research Center, Charité-Campus Buch, Berlin, Germany; 12 Charité Universitaetsmedizin Berlin, Campus Virchow Klinikum, Augustenburger Platz 1, Berlin, 13353 Germany

**Keywords:** Fingolimod, First dose, Electrocardiogram, Cardiac effects, Bradycardia, Atrioventricular conduction

## Abstract

**Background:**

First dose observation for cardiac effects is required for fingolimod, but recommendations on the extent vary. This study aims to assess cardiac safety of fingolimod first dose. Individual bradyarrhythmic episodes were evaluated to assess the relevance of continuous electrocardiogram (ECG) monitoring.

**Methods:**

START is an ongoing open-label, multi-center study. At the time of analysis 3951 patients were enrolled. The primary endpoints are the incidence of bradycardia (heart rate < 45 bpm) and second-/third-degree AV blocks during treatment initiation. The relevance of Holter was assessed by matching ECG findings with the occurrence of clinical symptoms as well as by rigorous analysis of AV blocks with regard to the duration of pauses and the minimal heart rate recorded during AV block.

**Results:**

Thirty-one patients (0.8%) developed bradycardia (<45 bpm), 62 patients (1.6%) had second-degree Mobitz I and/or 2:1 AV blocks with a lowest reading (i.e. mean of ten consecutive beats) of 35 bpm and the longest pause lasting for 2.6 s. No Mobitz II or third-degree AV blocks were observed. Only one patient complained about mild chest discomfort and fatigue. After 1 week, there was no second-/third-degree AV block.

**Conclusions:**

Continuous Holter ECG monitoring in this large real-life cohort revealed that bradycardia and AV conduction abnormalities were rare, transient and benign. No further unexpected abnormalities were detected. The data presented here give an indication that continuous Holter ECG monitoring does not add clinically relevant value to patients’ safety.

**Trial registration:**

NCT01585298; registered April 23, 2012.

## Background

Fingolimod (FTY720, brand name Gilenya®) has been approved for the treatment of relapsing remitting multiple sclerosis (RRMS). It exerts its therapeutic effects via modulation of sphingosine-1-phosphate (S1P-) receptors on lymphocytes, which results in the retention of circulating lymphocytes in the lymph nodes [1–4]. As a result, peripheral blood lymphocyte counts are reversibly reduced to approximately 30%, which is postulated to reduce recirculation of autoreactive lymphocytes and to prevent infiltration into the central nervous system [5, 6].

Receptors of this class, predominantly S1P1, are also expressed on atrial myocytes [7]. Fingolimod binding to S1P1 mediates a decrease in heart rate and prolongation of atrioventricular (AV) conduction. These effects are transient as fingolimod also induces internalization of these receptors, causing functional antagonism. Consequently, the negative chronotropic effects are limited to treatment initiation [6, 8–10].

A first-dose observation procedure is recommended. The exact procedure is defined by local product labels and differs between countries. It is generally required to observe patients for bradycardia by hourly measurements of pulse and blood pressure for at least six hours and to obtain an electrocardiogram (ECG) prior to dosing and at the end of the observation period. In contrast to the United States (US) and the Australian labels, the European Union (EU) label additionally recommends a continuous Holter ECG for six hours. In Japan, continuous ECG and observation of heart rate and blood pressure for 24 hours are recommended.

START is a prospective study designed to assess the cardiac safety of fingolimod first dose in detail and in a much larger cohort compared to previous studies. Patients and study sites were selected to closely resemble the broad range of fingolimod patients and treatment circumstances in daily practice. The study follows the EU label recommendations by obtaining a Holter ECG for six hours in addition to clinical monitoring and pre-/post first dose ECG. Therefore, START is the first interventional study that investigates in a large cohort whether continuous ECG monitoring detects clinically meaningful findings beyond pre-/post first dose ECG. The results might facilitate guidance on the degree of observation that is necessary to ensure patient safety.

## Methods

### Study design

START (NCT01585298) is an ongoing open-label, multi-center study in Germany in patients with RRMS receiving fingolimod at a daily dose of 0.5 mg. Its purpose is (a) to assess cardiac safety of fingolimod first dose with a special focus on bradycardia and AV conduction abnormalities, and (b) to analyze individual arrhythmic episodes more extensively in order to evaluate the clinical relevance of continuous ECG monitoring. Up to 7000 patients are planned to be enrolled based on a two-sided 95% confidence interval with a precision of 0.23%. Sample size calculation was based on the observed incidence of 0.963% of second-degree or higher AV blocks in the first 1000 patients enrolled. Annual interim analyses are planned per protocol to review whether the sample size needs to be adjusted. The results of the 2015 interim analysis are reported here. At the time of the interim analysis, the number of patients included has largely exceeded the sample size of previous studies, thereby adding significantly to the state of knowledge on the cardiac effects of fingolimod.

### Endpoints

The primary endpoints are the incidence of bradycardia, defined as heart rate < 45 bpm, and the incidence of second- and third-degree AV blocks during the six-hour monitoring period. For this analysis, three types of second-degree blocks were distinguished: Mobitz type I (progressive prolongation of the PR interval with the subsequent occurrence of a single non-conducted P wave, i.e. atrial impulse that creates a pause), 2:1 (second-degree block with a fixed 2:1 ratio of conducted and non-conducted P waves, that can be the result of either Mobitz I or Mobitz II type blocks) or Mobitz type II (constant PR interval followed by a non-conducted P wave, such that either an occasional dropped P wave or a regular conduction pattern of 2:1, 3:1, and so on is observed). Secondary endpoints include the incidence of other conduction abnormalities such as first-degree AV block (PR-interval prolongation), and QTc prolongation. The latter is defined as QTc > 450 ms in males and > 470 ms in females. Additionally, QTc prolongations > 500 ms are identified as these patients required overnight monitoring as specified by the EU label.

To analyze the incidence of bradycardia, pulse palpation by study personnel was used. In detail, study personnel were advised to measure the heart rate for at least 15 s. Second- and third-degree AV blocks were identified from continuous Holter ECG recordings. The remaining variables, i.e. PR- and QT-interval, were taken from 12-lead ECG data. Fridericia formula was used for correction of the QT intervals.

### Safety assessments

The incidence of adverse events (AE) and serious adverse events (SAE) was determined applying the definitions and standards of the ICH guideline on clinical safety data management. Further, the incidence of AE suggestive for cardiac events was determined using the following MedDRA preferred terms: Angina pectoris, chest discomfort, dizziness, dyspnoea, exertional dyspnoea, fatigue, palpitations, syncope, vertigo, positional vertigo, and blurred vision.

The incidence of overnight hospitalization was defined as the number of patients who experienced a cardiac AE (any AE out of the system organ class “cardiac disorders”) starting on the day of the first fingolimod intake and who were hospitalized for this event.

### Clinical relevance of continuous ECG monitoring

To assess the relevance of continuous ECG monitoring, the identified second-degree AV blocks were assessed for clinical relevance, i.e. the occurrence of any cardiac symptoms in these patients. Furthermore, continuous ECG recordings of patients with AV blocks were evaluated rigorously. The duration of pauses (RR intervals) and the minimal heart rate during second-degree AV blocks were determined post hoc by the cardiologist. The minimal heart rate during AV blocks was defined as the mean heart rate of 10 beats during an AV block to detect short lasting changes in heart rate. Holter ECG data of patients with cardiac symptoms were assessed for any abnormalities that might have predicted the symptoms. Alignment of evaluations of bradycardia as assessed by study nurse and the Holter ECG were also evaluated post hoc. No statistical comparison between Holter ECG and 12-lead ECG plus pulse palpation assessment with respect to the incidence of cardiac events was performed.

Results of START were analyzed descriptively. Where indicated, statistical tests were performed based on Student’s *t*-test unless otherwise noted.

### Approval and participants

The study protocol was approved by the respective state and institutional ethical standards committees at all participating sites (Competent ethics committee: Ethikkommission der Ärztekammer Nordrhein; EUDRACT No. 2012-000653-32-DE). Patients have to give written informed consent. The inclusion and exclusion criteria were defined to select patients with RRMS who were eligible for treatment with fingolimod (Gilenya®) according to the EU label. Patients were excluded if they received class Ia or III antiarrythmic drugs or beta blockers, heart-rate-lowering calcium channel blockers or other substances which may decrease heart rate (e.g. digoxin, anticholinesteratic agents or pilocarpine). Furthermore, patients were excluded if they have a history of second-degree Mobitz Type II or higher-degree AV block, Sick-sinus syndrome, Sino-atrial heart block, significant QT prolongation, symptomatic bradycardia or recurrent syncope, known ischemic heart disease, cerebrovascular disease, myocardial infarction, hypokalemia, congestive heart failure, cardiac arrest, uncontrolled hypertension, or severe sleep apnea.

### Study procedures

**Fig. 1:**
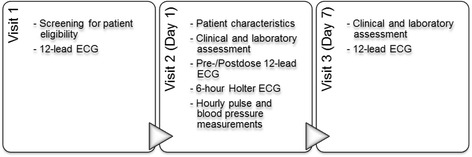
Visit schedule

Patients’ eligibility was determined during a 4-week screening phase (visit 1). At the first-dose visit (visit 2), patients underwent baseline assessments of basic demographic and relevant clinical characteristics including MS status, concomitant medication, vital signs, blood count and blood chemistry. A 12-lead ECG prior to the first dose of fingolimod was recorded. After the first intake of the study drug, patients were monitored for six hours by continuous Holter ECG recording, while heart rate and blood pressure were measured simultaneously, every hour. Afterwards, a second 12-lead ECG was recorded. The 6-hour monitoring period reflects the current EU label and was considered sufficient as the nadir of the heart rate decrease usually occurs four hours post-dose [9]. At visit 3, i.e. 1 week after study drug initiation, a third 12-lead ECG is recorded and vital signs, blood count and blood chemistry were assessed (Fig. 1). Adverse events were recorded at all visits.

If the patient’s heart rate at the end of the 6-hour period is the lowest following first dose administration, the monitoring had to be extended by at least two hours and until the heart rate rises. In those patients with evidence of clinically important cardiac events (e.g. persistent new-onset second-degree or third-degree AV block, heart rate < 45 bpm at six hours after first dose, QTc interval ≥ 500 msec), monitoring had to be extended until full resolution of symptoms, including overnight monitoring. If a patient required pharmacologic intervention during the first dose observation, the first dose monitoring strategy had to be repeated after the second dose of fingolimod. Study medication had to be interrupted or discontinued in cases of atypical neurological deterioration, lymphopenia < 200/μl, and a more than 5-fold elevation of liver function tests. In other cases the decision on continuation of study drug was at the discretion of the physician and the patient.

Each study site was equipped with the ECG-device CardioMem® CM3000-12, a 12-lead-continuous-ECG-digital recorder. The ECG recordings were pseudonymized and immediately transmitted via internet to the cardiology reading center for evaluation. The results of ECG analysis were available to the treating physician within less than 40 min. To identify AV blocks all Holter ECG recordings were assessed according to the same automated analysis process. Subsequently, two independent cardiologists manually reviewed every second- or third-degree AV block episode to determine the exact AV block subtype per episode, the number of episodes, the duration of the longest pause and the minimal heart rate (mean of ten consecutive beats).

Twice a year, an external and independent Data Safety Monitoring Board (DSMB), consisting of two neurologists and a cardiologist, reviewed safety-related issues. The DSMB was entitled to recommend changes in study conduct.

## Results

### Study patients and conduct

**Fig. 2:**
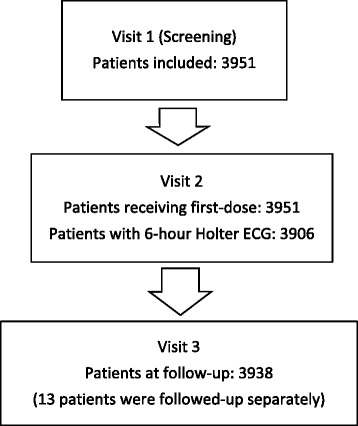
Patient disposition

The present analysis is based on 3951 patients recruited between June 2012 and January 2015. The majority of the patients were seen by office-based neurologists. Baseline demographics and MS history are presented in Tables 1 and 2. A Holter ECG was recorded at visit 2 in 3906 out of 3951 patients who received fingolimod (Fig. 1). 25 patients had discontinued medication after the first or second dose, and another 25 patients had discontinued the study drug further on (Table 3). In 38 patients (0.96%) adverse events were the primary reason for discontinuation. In the remaining 12 patients abnormal laboratory values or other reasons prompted to stop the study drug. 13 patients out of those who discontinued study drug did not show up for visit 3 (Fig. 2). These patients were followed-up separately confirming that they were alive and did not suffer from cardiac sequelae.

**Table 1:** Patient demographics and relevant baseline characteristics

	Fingolimod 0.5 mg (*N* = 3951)
Age groups in years, n (%)	
18–30	880 (22.3)
> 30–40	1222 (30.9)
> 40–55	1622 (41.1)
> 55–65	208 (5.3)
> 65	18 (0.5)
Sex	
Female, n (%)	2779 (70.3)
Duration of MS since first symptoms in years, mean ± SD	10.0 ± 7.6
Number of relapses in previous year, mean ± SD	1.6 ± 1.2
EDSS, mean ± SD	2.8 ± 1.6
DMT treatment within the last 6 months, n (%)	
No	827 (20.9)
Yes	3124 (79.1)

**Table 2:** Patient characteristics with potential relevance for cardiac events and frequency of symptoms by subgroup

	Overall population	Patients with bradycardia	Patients with second-degree AV block	Patients with AE	Patients with symptoms suggestive of cardiac events at visit 2	Patients who discontinued study drug due to AE
	*N* = 3951	*N* = 31	*N* = 62^a^	*N* = 1350	*N* = 120	*N* = 38
Demographics						
Age (years), mean ± SD	39.3 ± 10.4	42.1 ± 10.9	40.4 ± 11.7	39.3 ± 10.4	39.6 ± 10.4	42.3 ± 12.4
Female, n (%)	2779 (70.3)	14 (45.2)	57 (91.9)	1023 (75.8)	96 (80.0)	29 (76.3)
Concomitant medication known to prolong QT interval:						
SSRI n (%)	339 (10.1)	1 (3.2)	4 (6.5)	152 (11.3)	17 (14.2)	6 (15.8)
TCA n (%)	92 (2.3)	0	0	38 (2.8)	2 (1.7)	1 (2.6)
Amantadin n (%)	42 (1.1)	0	0	18 (1.3)	2 (1.7)	0
Carbamazepin n (%)	29 (0.7)	0	1 (1.6)	14 (1.0)	3 (2.5)	1 (2.6)
Fampridine n (%)	299 (7.6)	3 (9.7)	5 (8.1)	81 (6.0)	8 (6.7)	1 (2.6)
Heart rate at visit 2 pre-dose						
Heart rate (bpm), mean ± SD	73.9 ± 10.4	59.5 ± 8.0	75.0 ± 8.5	73.4 ± 10.4	73.3 ± 11.6	75.8 ± 10.6
Blood pressure at visit 2 pre-dose						
Systolic (mmHg), mean ± SD	121.8 ± 14.0	125.7 ± 20.3	117.5 ± 13.7	122.0 ± 14.1	123.9 ± 14.5	120.4 ± 15.4
Diastolic (mmHg), mean ± SD	78.5 ± 9.7	78.1 ± 13.1	75.7 ± 9.0	78.7 ± 9.8	79.9 ± 10.0	76.1 ± 11.1
Potassium levels at visit 2						
< 3.5 mmol/L	13 (0.3)	0	0	5 (0.4)	0	0
≥ 3.5–5.5 mmol/L	3853 (98.3)	31 (100)	61 (98.4)	1320 (98.5)	119 (99.2)	38 (100.0)
> 5.5 mmol/L	54 (1.4)	0	1 (1.6)	15 (1.1)	1 (0.8)	0
Symptoms suggestive of cardiac events^b^ during 6 h first-dose observation						
Patients with symptoms, n (%)	120 (3.0)	1 (3.2)^c^	1 (1.6)^d^	120 (8.9)	120 (100.0)	1 (2.6)

^a^One of the patients had both, bradycardia and second-degree AV block and is therefore included in both groups

^b^Cardiac symptoms are defined as the following MedDRA preferred terms: Angina pectoris, chest discomfort, dizziness, dyspnoea, dyspnoe exertional, fatigue, palpitations, syncope, vertigo, vertigo positional, blurred vision

^c^Fatigue and chest discomfort

^d^Palpitations

**Table 3:** Doses of study drug intake prior to discontinuation of study drug

	Fingolimod 0.5 mg *N* = 50
Number of capsules taken	n (%)
1	17 (34.0)
2	8 (16.0)
3–7	21 (42.0)
> 7	3 (6.0)
Missing	1 (2.0)

### Changes in heart rate

Patients receiving the first dose of fingolimod experienced on average a slight and transient decline in heart rate. 87.4% of the patients reached the nadir before the end of the 6-hour post-dose observation period. In 31 patients (0.8%) the heart rate dropped below 45 bpm. Holter ECG confirmed 23 of the 31 cases with a heart rate below 45 as measured by study personnel. For three patients no Holter was available und two other patients had an insufficient Holter ECG reading. Table 4 presents the heart beat dynamics of patients by subgroup, i.e. by time of the lowest heart rate measurement, by presence of bradycardia and by presence of AV blocks.

**Table 4:** Heart rate dynamics by subgroup within 6 h post-dose

	Number of patients	Pre-dose heart rate^a^ (bpm)	Lowest post-dose heart rate^a^ (bpm)	Maximum decline in heart rate^a^ (bpm)
	n (%)	Mean (range)	Mean (range)	Mean (SD)
	Fingolimod, *N* = 3951			
Overall population				
Overall population	3951 (100.0)	73.9 (45–132)	62.1 (31–101)	11.8 (8.47)
By time of lowest heart rate				
Patients with lowest heart rate at < 6 h	3455 (87.4)	73.8 (45–132)	61.9 (31–101)	11.9 (8.42)
Patients with lowest heart rate at 6 h	496 (12.6)	74.6 (48–114)	63.5 (38–90)	11.1 (8.76)
*p*-value^b^				0.0394
By presence of bradycardia				
Patients with bradycardia	31 (0.8)	59.5 (46–80)	41.6 (31–44)	17.9 (8.30)
Patients without bradycardia	3920 (99.2)	74.0 (45–132)	62.2 (45–101)	11.7 (8.45)
*p*-value^b^				0.0001
By presence of second-degree AV block				
Patients with second-degree AV block	62 (1.6)	75.0 (56–95)	62.7 (42–83)	12.2 (8.12)
Patients without second-degree AV block	3889 (98.4)	73.8 (45–132)	62.1 (31–101)	11.8 (8.48)
*p*-value^b^				0.691

^a^as measured by on-site study personnel

^b^Students *t*-test

### AV conduction abnormalities

**Fig. 3:**
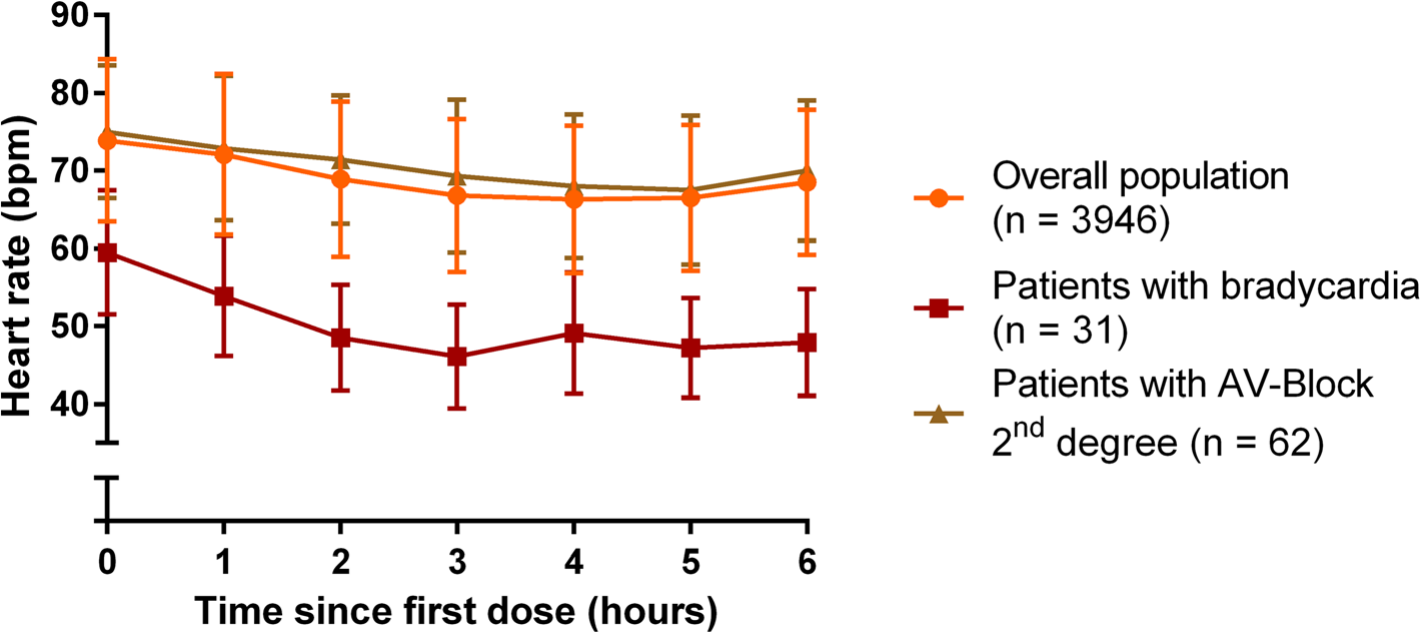
Changes in heart rate after the first dose of fingolimod. The time course of heart rate during the first 6 hours after fingolimod intake is presented for the overall population, patients with bradycardia and patients with second-degree AV block (absolute values and SD)

First-degree AV blocks (PR-interval > 200 ms in the ECG assessments) occurred in 2.7% of the patients before and in 5.8% of the patients after the first dose of fingolimod. In 62 patients (1.6%) a second-degree AV block was observed. Type 2:1 AV blocks were identified for 18 patients (Table 5). In 16 patients the 2:1 blocks emerged from Mobitz I blocks. In the two patients with 2:1 block not emerging from Mobitz I, the event was limited to a single episode. No Mobitz II second-degree AV blocks or third-degree AV blocks occurred (Table 5). On average the first second-degree AV blocks occurred 3.62 h after drug intake (SD 1.26, range 0.62 to 5.87). The mean minimal heart rate during second-degree AV block as assessed by Holter ECG (mean of ten consecutive beats) was 51.5 bpm (range 35 to 80 bpm) with no significant difference between the two types of second-degree AV blocks, Mobitz type I and 2:1 (*p* = 0.235). The longest pause observed in association with second-degree AV blocks was 2.6 s. The mean value was 1.9 s (SD 0.3), with no difference between 2:1 and Wenckebach-type AV blocks (*p* = 0.887). Heart rate dynamics of patients with second-degree AV blocks as measured by study personnel are shown in Fig. 3 and Table 4.

**Table 5:** Second- and third-degree AV blocks during treatment initiation

	Fingolimod 0.5 mg *N* = 3951
Patients with 12-lead ECG recording, n	*N* = 3951
Patients with first-degree AV block, n (%)	280 (7.1)
Pre-dose	96 (2.7)
Post-dose	206 (5.8)
Patients with Holter ECG recording, n	*N* = 3906
Patients with second-degree AV block, n (%)	62 (1.6)
Mobitz Type I (Wenckebach)	60 (1.5)^a^
2:1	18 (0.5)^a^
Mobitz Type II (Mobitz)	0
Patients with third-degree AV block, n (%)	0

^a^A patient might experience both Mobitz type I and 2:1 AV blocks during the six-hour monitoring

### Effects on repolarisation

The mean QTc interval in the overall population was 413.8 ms (range 352 to 470 ms) before and 417.7 ms (range 239 to 479 ms) after first dose. Applying > 450 ms in men and > 470 ms in women as a conservative threshold, only two men and four women had QTc intervals above their respective thresholds after fingolimod intake. None of these patients had symptoms suggestive of cardiac events and none required additional medication during first dose monitoring. All of these patients were continued on study medication. No patient had a QTc interval exceeding 500 ms, which would have warranted overnight monitoring as defined in the current EU label.

### Adverse events and cardiac symptoms

AEs were observed in 1350 patients (34.2%) and 117 patients (2.96%) experienced SAEs (Table 6). The most frequent types of AEs causing discontinuation of medication were cardiac, gastrointestinal, and nervous system disorders (Table 7). Symptoms reported as AE during the 6-hour monitoring suggestive of cardiac events occurred in 120 patients (3.04%, Table 6). Holter ECG revealed no rhythm abnormalities in these patients, as confirmed by the cardiologist. Only one case of symptomatic second-degree AV block (0.03%) and one case of symptomatic bradycardia (0.03%) were observed (Table 2). Neither syncope nor dyspnoea were observed in these patients. No patient required medical treatment for bradycardia or AV block. The patient with the lowest heart rate measured (31 bpm) was asymptomatic. In total, 42 patients (1.06%) were hospitalized overnight due to any cardiac adverse event that started during day 1 after first dose administration. Patient characteristics potentially increasing the risk for cardiac events by subgroup are presented in Table 2.

**Table 6:** Most frequent (serious) adverse events

	Fingolimod 0.5 mg *N* = 3951	
	Number of patients, n (%)	Number of events, n
Summary of adverse events		
Any adverse event	1350 (34.17)	2207
Any serious adverse event	117 (2.96)	152
Any adverse event leading to discontinuation of study drug	38 (0.96)	69
Common adverse events (>1% in SOC/PT)		
Nervous system disorders	403 (10.20)	464
Headache	199 (5.04)	205
Dizziness	72 (1.82)	73
MS relapse	58 (1.47)	59
General disorders and administration site conditions	240 (6.07)	265
Fatigue	135 (3.42)	138
Cardiac disorders	231 (5.85)	263
AV block first degree	69 (1.75)	70
AV block second degree	65 (1.65)	67
Bradycardia	45 (1.14)	45
Gastrointestinal disorders	217 (5.49)	246
Nausea	91 (2.30)	92
Diarrhoea	52 (1.32)	52
Infections and infestations	170 (4.30)	178
Nasopharyngitis	78 (1.97)	78
Investigations	130 (3.29)	150
Blood and lymphatic disorders	115 (2.91)	140
Lymphopenia	73 (1.85)	74
Musculoskeletal and connective tissue disorders	94 (2.38)	102
Skin and subcutaneous tissue disorders	66 (1.67)	69
Psychiatric disorders	61 (1.54)	63
Vascular disorders	60 (1.52)	61
Respiratory, thoracic and mediastinal disorders	59 (1.49)	60
Ear and labyrinth disorders	47 (1.19)	49
Common serious adverse events (>0.1% in SOC/PT)		
Cardiac disorders	52 (1.32)	65
AV block second-degree	31 (0.78)	33
Bradycardia	15 (0.38)	15
AV block	5 (0.13)	5
Nervous system disorders	33 (0.84)	36
MS relapse	18 (0.46)	19
General disorders and administration site conditions	7 (0.18)	7
Infections and infestations	7 (0.18)	7
Gastrointestinal disorders	6 (0.15)	8
Vascular disorders	6 (0.15)	6
Symptoms that might have resulted from cardiac events (at Visit 2)		
Fatigue	68 (1.72)	
Dizziness	26 (0.66)	
Chest discomfort	8 (0.20)	
Vertigo	7 (0.18)	
Palpitations	7 (0.18)	
Dyspnea	5 (0.13)	
Angina pectoris	3 (0.08)	
Blurred vision	2 (0.05)	
Syncope	1 (0.03)	
Exertional dyspnoe	1 (0.03)	

*PT* preferred term, *SOC* system organ class

**Table 7:** Study drug discontinuations due to adverse events

	Fingolimod 0.5 mg *N* = 38	
	Number of patients, n (%)	Number of events, n
Adverse events leading to discontinuation of study drug (<4% in SOC/PT)		
Cardiac disorders	16 (42.1)	20
AV block second-degree	9 (23.7)	10
Bradycardia	3 (7.9)	3
Gastrointestinal disorders	8 (21.1)	10
Nausea	4 (10.5)	4
Diarrhoea	3 (7.9)	3
Nervous system disorders	8 (21.1)	8
Dizziness	2 (5.3)	2
Headache	2 (5.3)	2
Vascular disorders	5 (13.2)	5
Hypertension	4 (10.5)	4
Investigations	5 (13.2)	5
QT prolongation	2 (5.3)	2
Skin and subcutaneous tissue disorders	5 (13.2)	5
Psychiatric disorders	4 (10.5)	4
General disorders and administration site conditions	3 (7.9)	4
Asthenia	2 (5.3)	2
Eye disorders	2 (5.3)	2
Vision blurred	2 (5.3)	2
Pregnancy, puerperium and perinatal conditions	2 (5.3)	2
Pregnancy	2 (5.3)	2

*PT* preferred term, *SOC* system organ class

### Follow up

At visit 3, first-degree AV blocks were found in 2.8% of patients. 12-lead ECG did not reveal any second- or third-degree AV blocks. However, since no continuous ECG was recorded at visit 3, accidental second- or third-degree AV blocks might have been missed. Bradycardia was present in only two patients with heart rates of 44 bpm (51 bpm at baseline) and 43 bpm (55 bpm at baseline). No symptoms were associated. Only two men and one woman had QTc intervals above the relevant thresholds (>450 and > 470 ms, respectively). None of the patients developed clinically relevant QTc interval prolongation (i.e. arrhythmias).

## Discussion

The data presented here demonstrate that 0.8% patients had bradycardia and 0.03% had symptomatic bradycardia with mild self-limiting symptoms. Transient second-degree AV blocks (Mobitz Type I and 2:1) were detected in 1.6% of patients. Bradycardia and AV block did not result in pre-syncope or syncope, demonstrating the benign and self-limiting nature of these cardiac effects. Despite the large size of the cohort and the transient occurrence of second-degree AV blocks Type I and 2:1, no Mobitz II or third degree AV blocks were observed. At visit 3, no patient had second- or third-degree AV blocks and the lowest heart rate was 43 bpm. The decrease in heart rate of patients with second-degree AV-blocks did not significantly differ from that in patients without such an event. Interestingly, the baseline mean heart rate of patients who developed bradycardia during first dosing was below 60, whereas in all other groups the mean baseline heart rate was above 70. This could be an indication that a patient with a low heart rate at onset may be at risk to develop substantial bradycardia.

Drug-related effects on myocardial repolarization (QT interval >450/470 ms) were observed in five patients, however, without a need for intervention. No patient required overnight monitoring due to QT interval prolongation > 500 ms, as warranted by the EU label. Although single reports on spontaneous arrhythmias have been published [11–16], no such events were observed in this large cohort.

The results of START are consistent with previous studies. Incidence of bradycardia and second-degree AV blocks in previous studies were 1.3 to 1.4% (bradycardia), 1.3 to 2.6% (Mobitz type I second-degree AV block, Wenckebach) and 0.5 to 1.4% (2:1 second-degree AV block) [7–10]. In a cohort of 625 healthy volunteers, transient type I second-degree AV blocks (Wenckebach) were observed in 14 (2.2%) individuals during nighttime. In three cases, AV blocks were also observed during daytime [17]. The occurrence of AV blocks beyond the 6-hour observation interval including nocturnal events were investigated in the FREEDOMS II trial [8]. Fingolimod-induced second-degree AV blocks usually occurred in the first six hours. The data indicated that nighttime second-degree AV blocks were infrequent and occurred in 0.6% of the patients receiving fingolimod and in 2.0% of the placebo patients. These results underpin the interpretation that the incidence of AV blocks during nighttime (>12 h after first dose) does not seem higher than in the healthy population. However, as the study cohorts differ, a valid comparison of incidence rates would require adjustment for variations in patient characteristics.

Except for one case accompanied by mild symptoms, AV blocks observed in the START study were not associated with cardiac symptoms. Thus, the pathologic ECG findings were usually not associated with any clinically relevant event. Conversely, none of the few mild cardiac symptoms observed showed a corresponding event in the Holter ECG. In summary, Holter ECG data provide scientifically valuable information of an in-depth understanding of the electrocardiographic effects of fingolimod, but does not help to identify patients at risk for clinically relevant cardiac symptoms.

As lower degree AV conduction abnormalities also occur in an otherwise healthy population without clinical significance, they are generally not considered harmful [18]. In general, type I second-degree AV blocks do not require intervention as long as they are asymptomatic [19]. On the contrary, third-degree and type II second-degree AV blocks usually warrant intervention and even constitute an indication for permanent pacemaker implantation, which indicates their prognostic relevance [19]. However, this approach does not apply to drug-induced lower degree AV blocks, which are associated with clinical concerns as they might predict more severe AV conduction disturbances [18]. Drugs like beta-blockers or calcium channel blockers are known for their potential to induce deleterious AV conduction abnormalities [18]. These disturbances can even relapse after discontinuation of the inducing drug and may require permanent use of pacemakers [20, 21].

The characteristics of second-degree AV blocks in the START study indicate that the nature of AV conduction effects of fingolimod might differ from other AV block inducing drugs. For example, drug-induced AV blocks are usually associated with significant slowing of heart rate. However, in fingolimod-induced second-degree AV blocks no relevant decrease in heart rate was observed. The mean minimal heart rate over ten consecutive beats during AV block observed was 51.5 bpm. Furthermore, the Holter ECGs did not show relevant pauses. Usually, pauses are reported if they exceed 3 s in patients with sinus rhythm. The longest pause recorded in association with a second-degree AV block in this study was 2.6 s. The absence of relevant pauses is underlined by the fact that the events mostly remained asymptomatic. Finally, none of the AV blocks progressed to a complete block (third-degree AV block), as would have been expected for drugs known to induce AV blocks (e.g. calcium channel blockers).

Therefore, AV blocks associated with fingolimod treatment initiation can be considered as rather benign, self-limiting and associated with an either low or virtually non-existing propensity to progression to a complete AV block. Although affected by fingolimod, overall AV conduction, which is determined by several ion currents (i.e. particularly by calcium currents) [22, 23], remains preserved. In the present study, AV blocks were not preceded by excessive slowing of heart, what would be expected, but followed increases in heart rate. This suggests that slowing of AV conduction is due to a positive rate-dependent electrophysiological effect of fingolimod. A mild direct effect of fingolimod on the AV node was postulated in a rodent study, where S1P1 receptor mRNA expression was found to be high in the AV node. Fingolimod prolonged the cycle length in isolated AV node cells by 9% from 230 to 251 ms. Under pathological conditions (such as ischemia/reperfusion), fingolimod did not further prolong AV conduction. Fingolimod-induced AV node conduction abnormalities were discussed to be the result of a direct effect of the drug on ion channels expressed in the AV node with mild prolongation of AV conduction time [24].

Generally speaking, the effects of fingolimod on AV conduction have to be interpreted and weighed in the broader context of its beneficial effects in a progressive disease such as MS [1–3].

## Conclusions

The results presented here are derived from an interim analysis. The events identified by continuous ECG monitoring were associated neither with severe clinical symptoms nor with clinically relevant pauses or clinically relevant drops in heart rate. Furthermore, patients with clinical symptoms had no pathological findings on Holter ECG. Cardiac symptoms, if present, were rare, mild, and occurred independently of the expected benign AV conduction abnormalities and heart rate decline. ECG monitoring did not detect any otherwise unforeseen arrhythmias. In summary, clinically relevant cardiac events were not associated with abnormalities on continuous ECG monitoring and conduction abnormalities as demonstrated by Holter ECG usually remained clinically asymptomatic. Even without statistical evidence, the results presented here suggest that continuous Holter ECG monitoring after fingolimod first dose likely do not add clinically relevant value to patient safety with respect to bradycardia or conduction abnormalities in a general RRMS population without cardiovascular comorbidities.

## Abbreviations

AE: Adverse event
AV: Atrioventricular conduction
DSMB: Data Safety Monitoring Board
ECG: Electrocardiogram
EDSS: Expanded Disability Status Scale
EU: European Union
EUDRACT: European Clinical Trials Database
FTY720: Fingolimod
RRMS: Relapsing remitting multiple sclerosis
S1P: Sphingosine-1-phosphate
SAE: Serious adverse event
SD: Standard deviation
US: United States
